# Histological Comparison in Rats between Carbonate Apatite Fabricated from Gypsum and Sintered Hydroxyapatite on Bone Remodeling

**DOI:** 10.1155/2015/579541

**Published:** 2015-10-04

**Authors:** Yasunori Ayukawa, Yumiko Suzuki, Kanji Tsuru, Kiyoshi Koyano, Kunio Ishikawa

**Affiliations:** ^1^Section of Implant and Rehabilitative Dentistry, Division of Oral Rehabilitation, Faculty of Dental Science, Kyushu University, 3-1-1 Maidashi, Higashi-ku, Fukuoka 812-8582, Japan; ^2^Department of Biomaterials, Faculty of Dental Science, Kyushu University, 3-1-1 Maidashi, Higashi-ku, Fukuoka 812-8582, Japan

## Abstract

Carbonate apatite (CO_3_Ap), the form of apatite found in bone, has recently attracted attention. The purpose of the present study was to histologically evaluate the tissue/cellular response toward the low-crystalline CO_3_Ap fabricated using a dissolution-precipitation reaction with set gypsum as a precursor. When set gypsum was immersed in a 100°C 1 mol/L Na_3_PO_4_ aqueous solution for 24 h, the set gypsum transformed into CO_3_Ap. Both CO_3_Ap and sintered hydroxyapatite (s-HAp), which was used as a control, were implanted into surgically created tibial bone defects of rats for histological evaluation. Two and 4 weeks after the implantation, histological sections were created and observed using light microscopy. The CO_3_Ap granules revealed both direct apposition of the bone matrix by osteoblasts and osteoclastic resorption. In contrast, the s-HAp granules maintained their contour even after 4 weeks following implantation which implied that there was a lack of replacement into the bone. The s-HAp granules were sometimes encapsulated with fibrous tissue, and macrophage polykaryon was occasionally observed directly apposed to the implanted granules. From the viewpoint of bone remodeling, the CO_3_Ap granules mimicked the bone matrix, suggesting that CO_3_Ap may be an appropriate bone substitute.

## 1. Introduction

The golden standard for the reconstruction of bone defects is believed to be an autograft because it demonstrates osteoconduction, osteoinduction, and osteogenesis without causing any immunological response [1–4]. However, surgical intervention of the host site to harvest the bone that is required for an autograft is a serious drawback [5]. Moreover, the amount of collectable bone is limited, particularly in the case of dental treatment. Therefore, bone defects are usually augmented using artificial bone substitutes in combination with or without an autograft.

At present, sintered hydroxyapatite [s-HAp: Ca_10_(PO_4_)_6_(OH)_2_] and sintered *β*-tricalcium phosphate [*β*-TCP: Ca_3_(PO_4_)_2_] are often used in dentistry. Although the inorganic component of bone mainly comprises apatite, it is not the highly crystalline form HAp but the low-crystalline carbonate form CO_3_Ap [6]. s-HAp has been used in clinical applications instead of CO_3_Ap because CO_3_Ap thermally decomposes at the high temperature required for sintering. However, we propose a fabrication method of CO_3_Ap blocks by a compositional transformation reaction based on the dissolution-precipitation reaction using a precursor. In this reaction, a calcite block that was fabricated by exposing Ca(OH)_2_ compact to CO_2_ is immersed in a phosphate salt solution [7]. When calcite is immersed in an aqueous solution, calcite dissolves and supplies Ca^2+^ and CO_3_^2−^, as shown in (1). No further reaction will occur if water contains no other ions. However, if the solution contains phosphate salt, the solution is supersaturated with respect to CO_3_Ap. Therefore, Ca^2+^, PO_4_^3−^, and CO_3_^2−^ precipitate as CO_3_Ap, as shown in (2).

(1) $$\begin{array}{c} {CaCO}_{3}⟶{Ca}^{2+}+{{CO}_{3}}^{2-} \end{array}$$

(2) $$\begin{array}{c} {{Ca}^{2+}+{{PO}_{4}}^{3-}+{{CO}_{3}}^{2-}+OH}^{-}⟶{Ca}_{10-a}{\left({PO}_{4}\right)}_{6-b}{\left({CO}_{3}\right)}_{c}{\left(OH\right)}_{2-d} \end{array}$$

Based on this dissolution-precipitation reaction, calcite transforms to CO_3_Ap, maintaining its macroscopic structure.

On the other hand, gypsum has a self-setting property. Therefore, CO_3_Ap in any shape can be fabricated when set gypsum is used as a precursor. The gypsum dissolves in water to supply Ca^2+^, as shown in

(3) $$\begin{array}{c} {CaSO}_{4}⟶{Ca}^{2+}+{{SO}_{4}}^{2-} \end{array}$$

If the solution contains PO_4_^3−^ and CO_3_^2−^, the solution is supersaturated with respect to CO_3_Ap; therefore, Ca^2+^, PO_4_^3−^, and CO_3_^2−^ would be precipitated as CO_3_Ap (see (2)) that is similar to the case when calcite is used as a precursor. In the reaction, CO_3_^2−^ can be supplied by adding carbonate salt to the phosphate salt solution [8] or from the atmosphere, particularly when the phosphate salt solution is alkaline.

CO_3_Ap fabricated in this manner is thought to be a promising artificial bone substitute. However, few histological examinations have been conducted for CO_3_Ap, and no histological examination was reported for CO_3_Ap fabricated using set gypsum as a precursor.

Therefore, the objective of this study was to investigate the tissue response to CO_3_Ap fabricated from set gypsum using s-HAp as a control.

## 2. Materials and Methods

### 2.1. CO_3_Ap Granules

B-type carbonate apatite (CO_3_Ap) granules were prepared using set gypsum as a precursor that is similar to the method previously described [9]. First, set gypsum was fabricated by mixing calcium sulfate hemihydrate (CaSO_4_·1/2H_2_O, Wako Pure Chemical Industries, Kyoto, Japan) and distilled water at a water-to-powder ratio of 0.5. After the gypsum paste was allowed to set at room temperature for 24 h, it was crushed and sieved to obtain 200 to 400 *μ*m granules.

Further, the gypsum granules were immersed in a 1 mol/L Na_3_PO_4_ (Wako Pure Chemical Industries) aqueous solution at 100°C for 24 h.

### 2.2. Sintered HAp Granules

s-HAp was fabricated by sintering commercially obtained hydroxyapatite powder (HAP200; Taihei Chemical, Osaka, Japan). HAp powder (0.2 g) was placed in a stainless steel mold and uniaxially pressed with an oil pressure press machine (Riken Power; Riken Seiki, Niigata, Japan) under 5 MPa. HAp compacts were heated in an electronic furnace at 5°C/min until 1200°C and sintered at that temperature for 4 h and then cooled inside the furnace. s-HAp was crushed and sieved to obtain 200 to 400 *μ*m granules.

### 2.3. Characterization

Composition and the crystallite size of the specimens were determined using an X-ray diffractometer (XRD: D8 Advance, Bruker AXS GmbH, Karlsruhe, Germany) operated at 40 kV and 40 mA. The diffraction angle was continuously scanned from 10° to 60° in 2*θ* at a scanning rate of 2°/min. Specific surface area of both granules was measured using Brunauer-Emmett-Teller method utilizing adsorption of nitrogen gas using a surface area analyzer (Gemini 2375, Micromeritics, Norcross, GA, USA). Furthermore, Fourier transformed infrared (FT-IR) spectroscopy analysis was performed with an FT-IR spectrometer (Spectrum 2000LX; Perkin-Elmer Co. Ltd., Kanagawa, Japan), and results were recorded in the wave number range of 370–7800 cm^−1^. Then, the CO_3_ content in the apatitic structure was estimated using the method reported previously [10]. The porosity of both materials was calculated based on the bulk density of specimens and the density of HAp and expressed as a percentage as shown in the following equation:

(4) $$\begin{array}{c} Porosity=100\times \frac{d_{HAp}-d_{specimen}}{d_{HAp}}, \end{array}$$

where *d*_specimen_ and *d*_HAp_ are the apparent density of specimen and theoretical density of HAp (3.16 g/cm^3^).

Also, morphological observations of both granules were performed using scanning electron microscopy under an accelerating voltage of 15 kV (S-3400N, Hitachi High-Technologies, Tokyo, Japan).

### 2.4. Surgical Procedure

Forty-eight 8-week-old male Sprague Dawley rats (each weighing approximately 200–250 g) were used in this study. In this study, Guide for the Care and Use of Laboratory Animals, National Research Council, USA, was strictly observed and all animal experiments were conducted under the approval of the ethical committee of animal experimentation of the university (approval number: A24-221-0).

Under systemic anesthesia, the tibial bones of both legs were carefully exposed by exfoliation. After dissection of the periosteum, artificial bone cavities of 1.5 × 5.0 mm were prepared in the cortical bone of both tibiae by drilling with a round bur attached to a dental handpiece [11]. The bone cavity was reconstructed up to the level of the previous bone surface with granules that were sterilized in an autoclave at 105°C for 60 min. The skin flap was then closed with suturing. After the surgery, buprenorphine (Lepetan, Otsuka Pharmaceutical, Tokyo, Japan) was injected as an analgesic.

### 2.5. Histological Procedure

Two and 4 weeks after implantation, the animals were euthanized and fixed with a solution containing 4% paraformaldehyde and 5% glutaraldehyde in 0.1 mol/L phosphate buffer at pH 7.4. Tibial bones were carefully exposed by exfoliation and extracted and then immersed in the same fixative for 1 week at room temperature. After decalcification with Plank-Rychlo solution [12] for 24 h, specimens were dehydrated through a graded series of ethanol and N-butyl glycidyl ether followed by embedding in an epoxy resin (Quetol 651; Nisshin EM, Tokyo, Japan). The specimens were sectioned in 1 *μ*m thick slices using an ultramicrotome (ULTRACUT S; Reichert-Nissei, Tokyo, Japan), stained with toluidine blue, and then examined under light microscope for the detection of grafted material and new bone formation.

## 3. Results

**Figure 1:**
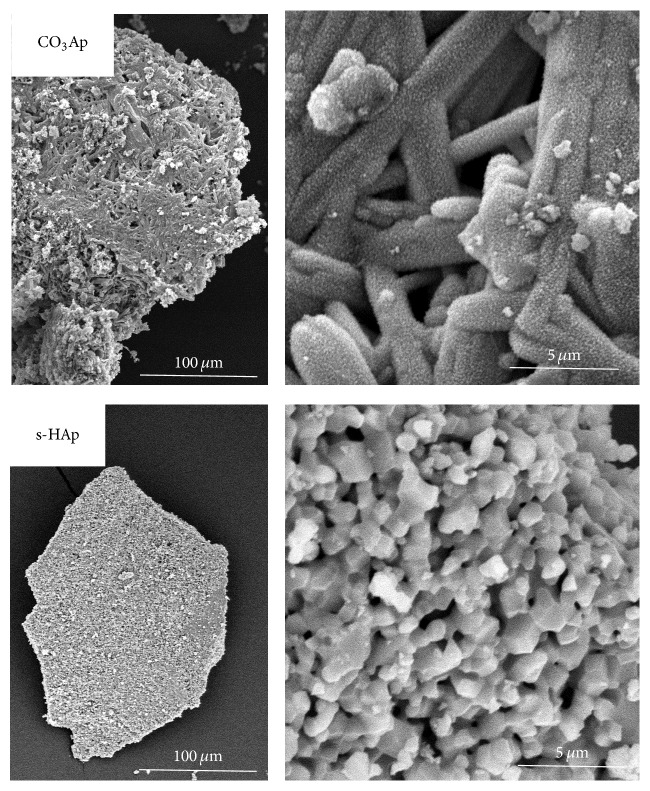
Scanning electron microscopic images of CO_3_Ap (upper) and s-HAp (lower) granules. Original magnification: ×270 (left), ×5000 (right).

Figure 1 shows the SEM images of both CO_3_Ap and s-HAp granules. The density was somewhat higher in s-HAp granules. In case of CO_3_Ap granule, morphologies of needle-like gypsum crystal structure remained, which were covered with many fine granular crystals. On the surface of s-HAp granule, typical grain structure was observed. Other characteristics of both CO_3_Ap and s-HAp granules are summarized in Table 1.

**Figure 2:**
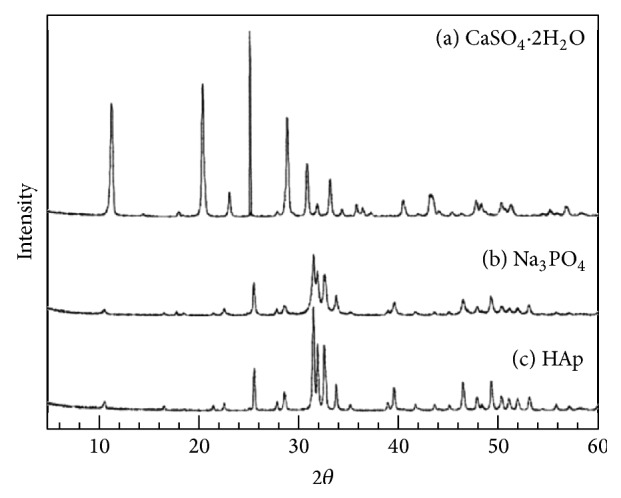
Powder XRD patterns of set gypsum before (a) and after (b) treatment in 1 mol/L Na_3_PO_4_ solution at 100°C for 24 h and of sintered hydroxyapatite (c).

Figure 2 summarizes the powder XRD patterns of the granules used in this study. Set gypsum granules were found to be CaSO_4_·2H_2_O (Figure 2(a)), and set gypsum immersed in Na_3_PO_4_ solution demonstrated a broad apatitic peak, indicating that it had undergone a compositional transformation from gypsum to low-crystalline apatite (Figure 2(b)). In contrast, the s-HAp granules revealed a sharp peak typical for high crystalline HAp (Figure 2(c)).

**Figure 3:**
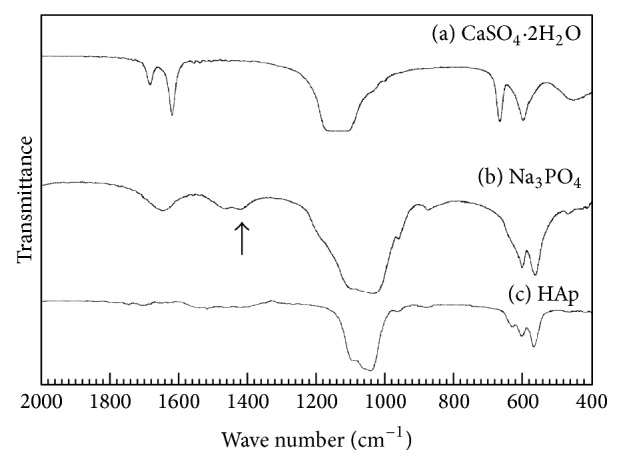
FT-IR spectra of (a) set gypsum, (b) gypsum after treatment in 1 mol/L Na_3_PO_4_ solution at 100°C for 24 h, and (c) sintered hydroxyapatite. The arrow indicates the additional absorption band assigned to a stretching vibration of CO_3_^2−^ of B-type carbonate apatite, in which the PO_4_^3−^ lattice site is substituted by CO_3_.

Figure 3 summarizes the FT-IR spectra of the granules used in this study. Set gypsum granules demonstrated absorption bands in three wave number regions: 1700–1600 cm^−1^, 1300–1000 cm^−1^, and 700–600 cm^−1^ (Figure 3(a)). The set gypsum immersed in Na_3_PO_4_ solution revealed apatitic peaks along with a carbonate peak typical for B-type carbonate apatite in which CO_3_^2−^ is replaced with PO_4_^3−^. The CO_3_ content in the apatitic structure was estimated as 1.4 mass% from the FT-IR spectra. However, the s-HAp granules revealed an apatitic peak but no peaks corresponding to CO_3_^2−^ (Figure 3(c)).

**Figure 4:**
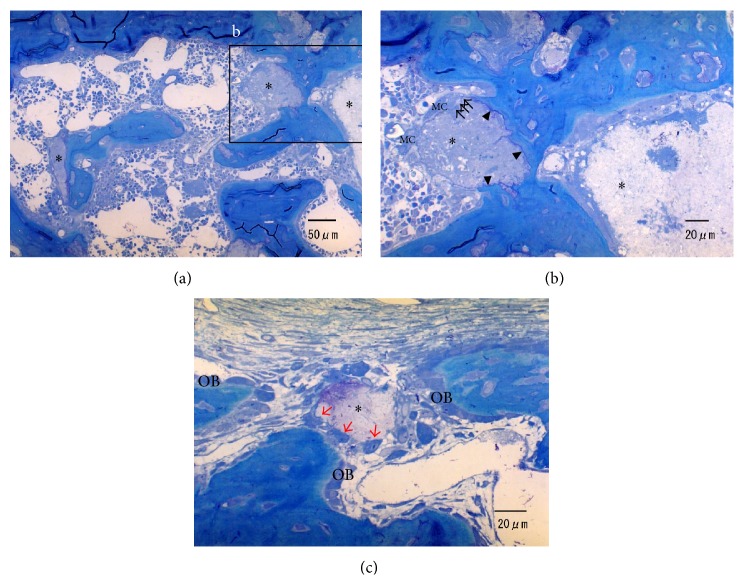
Histologic sections of defects filled with CO_3_Ap (*∗*) after 2 weeks of implantation. (a, b) Direct bone deposition to CO_3_Ap is detected (arrowheads). Howship's lacunae (black arrows) formed by multinucleated cells (MC), probably osteoclasts, are observed around CO_3_Ap. (c) There are some cells (red arrows) that have a similar morphology to osteoblasts (OB) around CO_3_Ap.

Figure 4 summarizes the histological pictures of CO_3_Ap granules 2 weeks after implantation. The CO_3_Ap granules were almost circumscribed by the newly formed bone (Figure 4(a)). The contact between the new bone and CO_3_Ap was intimate and direct without any intervention of soft tissue. Direct contact of cells, probably osteoblasts and osteoclasts, was observed on some surfaces of the CO_3_Ap granules (Figures 4(b) and 4(c)).

**Figure 5:**
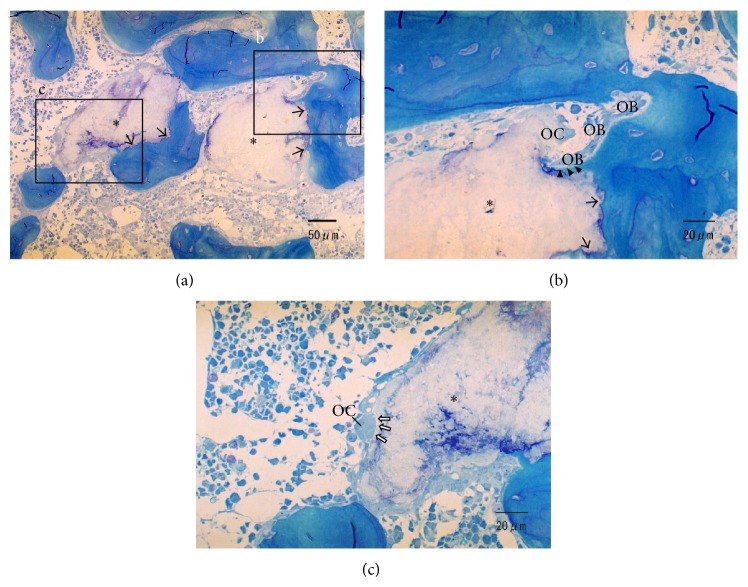
Histologic sections of defects filled with CO_3_Ap (*∗*) 4 weeks after implantation. (a, b) Direct contact with new bone to CO_3_Ap is observed, and the outline of CO_3_Ap is somewhat irregular (black arrows). (b) Remodeling of the material, which is engaged by the coupling of osteoblastic (OB) and osteoclastic cells (OC), is observed. Direct deposition of the bone matrix onto CO_3_Ap by osteoblasts is indicated (arrowheads). (c) Howship's lacuna (blank arrows) formed by osteoclast is observed around the CO_3_Ap.

Figure 5 summarizes the histological pictures of the CO_3_Ap granules 4 weeks following implantation. At 4 weeks, some granules were in direct contact with the new bone (Figures 5(a) and 5(b)); however, otherwise the surface of the granules was surrounded by a number of osteoclastic cells (Figure 5(c)). Direct deposition of the bone matrix onto the CO_3_Ap granules by osteoblasts was observed (Figure 5(b)). Consequently, the newly formed bone at the implanted area, including the CO_3_Ap granules, appeared to undergo normal bone remodeling.

**Figure 6:**
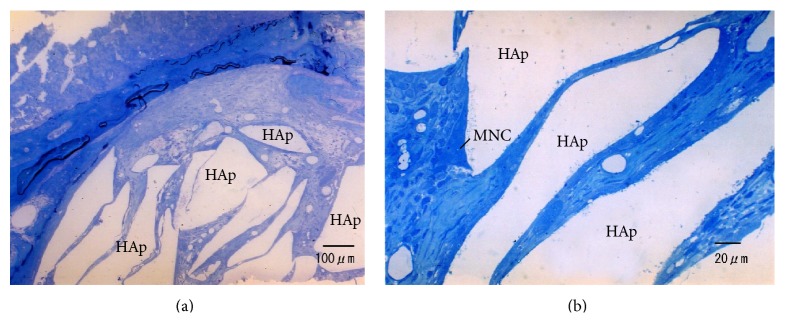
Histologic sections of defects filled with s-HAp 2 weeks after implantation. (a) Most surfaces of HAp granules are surrounded by connective tissue. (b) A multinucleated cell (MNC) is observed around the s-HAp granules. This MNC is characterized by a uniform cytosolic stain with a smaller number of endosomal structures.

Figure 6 summarizes the histological pictures of s-HAp 2 weeks after implantation. The s-HAp granules were surrounded by connective tissue 2 weeks after implantation (Figure 6(a)). Some multinucleated giant cells (MNCs), possibly macrophage polykaryon, were directly apposed to the s-HAp granules. These giant cells revealed a uniform stainability of cytoplasm with a number of nuclei, and their size was >50 *μ*m. Therefore, these cells were thought to be foreign body giant cells and not osteoclasts (Figure 6(b)).

**Figure 7:**
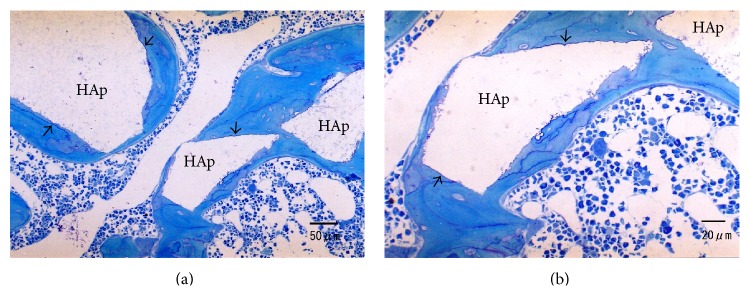
(a, b) Histologic sections of defects filled with s-HAp 4 weeks after implantation. Direct bone contact to s-HAp is observed (arrows). The outline of the s-HAp granules did not change in comparison with those after 2 weeks following implantation.

At 4 weeks following implantation, the boundary between the s-HAp granules and surrounding bone was clearly distinguishable (Figure 7(a)). The amount of new bone formed at 4 weeks after implantation was larger than that after 2 weeks. The s-HAp granules retained their original size and shape (Figure 7(b)).

No inflammatory reaction that was characterized by the migration of immunocompetent cells was observed around the implanted granules regardless of their composition. All granules demonstrated excellent tissue response.

## 4. Discussion

A clear difference was observed between the CO_3_Ap and s-HAp granules with respect to osteoconductivity. New bone was formed around the CO_3_Ap granules without fibrous tissue as early as 2 weeks after implantation. Moreover, osteoblastic cells and bone matrix deposition were found on the surface of the CO_3_Ap granules at 2 weeks after implantation. In contrast, the s-HAp granules were surrounded by fibrous tissue at this stage. Therefore, no direct contact between osteoblasts and s-HAp was observed at this stage. Although the s-HAp granules were also bonded to the bone, these differences demonstrated that CO_3_Ap had higher osteoconductivity than s-HAp. These results are consistent with a previously reported cell study, although the precursor we employed is different. Nagai et al. used human bone marrow cells (hBMCs) and evaluated the effect of CO_3_Ap on cell response. It was demonstrated that hBMCs incubated on CO_3_Ap demonstrated a much higher expression of osteoblastic markers of differentiation, such as type I collagen, alkaline phosphatase, osteopontin, and osteocalcin, than hBMCs incubated on s-HAp [13].

Furthermore, a clear difference was observed between CO_3_Ap and s-HAp with respect to their resorption capability. In case of CO_3_Ap, MNCs with Howship's lacunae were observed around the CO_3_Ap granules at all stages. The morphology of these cells was very similar to that of osteoclasts at the bone remodeling front. In contrast, for the s-HAp granules, multinucleated and extremely large cells with a dimension of >50 *μ*m were observed at an early stage. These cells produced no resorption lacuna and their morphology was not similar to that of osteoclasts that are characterized by the abundant endosome. Instead, the cells resembled the macrophage polykaryon.

The mechanism of calcium phosphate resorption is still under debate. Dersot et al. reported that s-HAp was not resorbed by osteoclasts; however, it was resorbed by MNCs, which did not have tartrate-resistant acid phosphatase activity (a typical marker for osteoclasts); instead, it had a nonspecific esterase (a typical macrophage marker) [14]. Wada et al. reported that *β*-TCP was not resorbed with osteoclasts; however, it was resorbed by MNCs. No ruffled border was observed, and acid phosphatase was easily inactivated by tartrate [15]. On the contrary, several researchers reported that calcium phosphates were resorbed by osteoclasts. Baslé et al. reported that both biphasic calcium phosphate and bovine bone apatite were resorbed by osteoclasts, and bovine bone apatite was resorbed by osteoclasts [16]. Yuasa et al. reported that low-crystalline apatite that was fabricated by setting reaction of apatite cement was resorbed by osteoclasts [17]. It should be noted that the mechanism of calcium phosphate resorption could be different in different studies. The composition of calcium phosphates and the crystallinity are different. However, the fact that these characteristics differ may present the opportunity for a comparative study in the future.

Our s-HAp results were consistent with the findings of previous papers in that MNCs were observed around the s-HAp granules. Macrophage polykaryon is also known to create resorption pits. However, the pits tend to be very shallow and small [18]. This implies that the extracellular degradation activity of macrophage polykaryon is relatively low. In addition, the dissolution rate of high crystalline materials, including s-HAp, is small [1, 19, 20]. Because of the lower resorption ability of macrophage polykaryon and because s-HAp has a low dissolution rate, MNCs cannot dissolve s-HAp. Therefore, the outline of s-HAp was preserved even 4 weeks after implantation.

Appearance of osteoblastic cells around the granules of *β*-TCP has been reported to always be followed by the resorption of the material by MNCs [15]. In the present study, s-HAp was juxtaposed by MNCs in the early stage, and the granules were encapsulated by the new bone at the later stage. Therefore, it is possible that the juxtaposition of MNCs to s-HAp in the early stage plays a role in the subsequent bone formation.

In summary, unlike conventional s-HAp, the newly developed CO_3_Ap demonstrated bone-like remodeling characteristics in the bone cavity, such as osteoblastic direct bone formation and osteoclastic resorption. These traits are thought to be critical for a bone substitute material. However, the limitation of the present study is that we employed a rodent small-defect model. In an actual bone augmentation procedure, a large volume of bone graft material is applied to the desired region. In the present study, the cellular response toward the material could be clarified; however, when the material is applied in a large volume, cell penetration and/or vascularization among the graft material granules may play a critical role in the success of bone augmentation. In future studies, the actual bone augmentation capability of CO_3_Ap, such as in alveolar bone preservation or in large defect filling, should be elucidated.

## 5. Conclusions

Low-crystalline CO_3_Ap revealed higher osteoconductivity when compared with high crystalline s-HAp. CO_3_Ap granules demonstrated both resorption by osteoclastic cells and direct bone formation by osteoblastic cells, and these characteristics are similar to that of the bone. Thus, CO_3_Ap granules are an ideal artificial bone substitute. Further studies are required based on the results obtained in this study.

## Figures and Tables

**Table 1:** Crystallite size, specific surface area, and the porosity of the specimen.

	Crystallite size (nm)	Specific surface area (m^2^/g)	Porosity (%)
CO_3_Ap	51.1	48.5	64.2
s-HAp	173.2	0.7	54.2
